# P-1230. Dose-Escalation Study to Evaluate the Pharmacokinetics and Safety of Single and Repeat Doses of Ceftibuten in Healthy Participants

**DOI:** 10.1093/ofid/ofae631.1412

**Published:** 2025-01-29

**Authors:** Mary Beth Dorr, Carlos Fernando de Oliveira, Kathryn Lowe, Gregory A Winchell, Paul McGovern

**Affiliations:** Venatorx, Malvern, Pennsylvania; Venatorx Pharmaceuticals, Inc, Malvern, Pennsylvania; Venatorx Pharmaceuticals, Malvern, Pennsylvania; Winchell Pharma Consulting LLC, Norristown, Pennsylvania; Venatorx Pharmaceuticals, Malvern, Pennsylvania

## Abstract

**Background:**

Venatorx is developing ledaborbactam etzadroxil (LED-E), the prodrug of the active β-lactamase inhibitor ledaborbactam, in combination with ceftibuten (CTB), to address an unmet medical need for new oral (PO) agents to treat serious infections caused by drug resistant pathogens. Cis-CTB is the highly active isomer of CTB. The purpose of this study was to evaluate the pharmacokinetics (PK) and safety of CTB in healthy adult subjects at potential doses to be studied in combination with LE.

**Figure:**
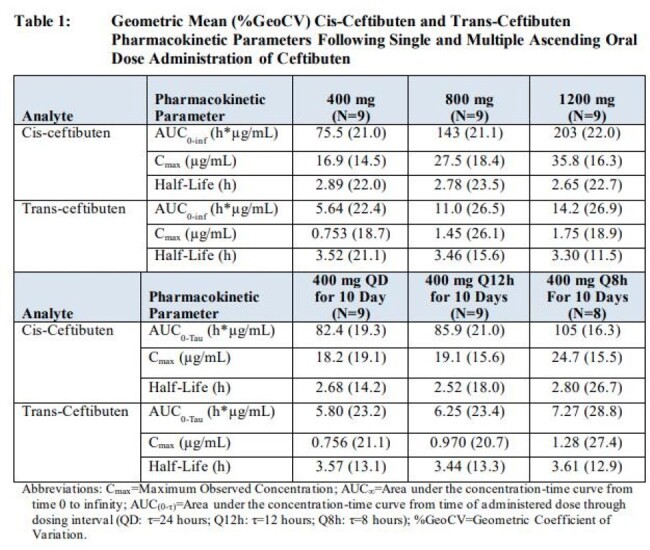

**Methods:**

Thirty-six participants (PT), 12 per cohort (CTB 9, placebo, PBO 3) received a single PO dose of CTB (400, 800, or 1200mg) or matched PBO on Day 1. Following a one-day washout period, the same PT received repeat PO doses of CEF (400mg daily, 400mg q12h, or 400mg q8h) for 10 days. Serial blood samples and urine were collected for determination of single dose and steady-state plasma PK and urinary excretion of cis- and trans-CTB. Safety was assessed by recording adverse events, and changes in safety laboratory, vital signs, and ECGs.

**Figure:**
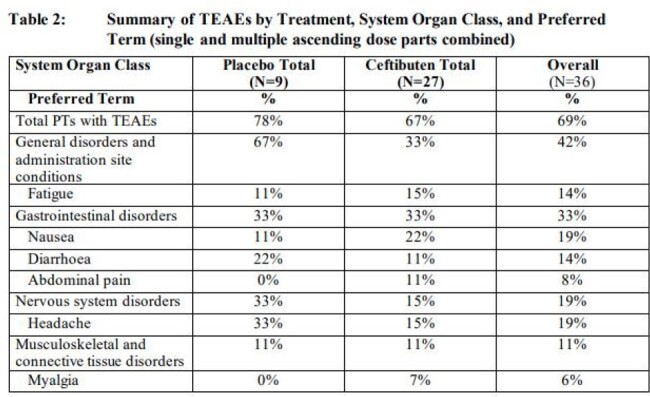

**Results:**

Geometric mean (%CV) cis- and trans-CTB PK parameters for single and multiple doses are shown in Table 1. AUC_(0-τ)_ and AUC_∞_, appeared to increase in a dose proportional manner, while maximum concentrations (Cmax) appeared to increase in a less than dose proportional manner across the entire dose range in the single dose part of the study. Low levels of accumulation of cis-CTB were observed at steady state, with the highest accumulation ratio of 1.24 in the 400mg q8h cohort. Cis-CTB and trans-CTB recovery in urine was 47% and 6% following a 1200mg single dose, respectively.

The incidence of treatment-emergent adverse events (TEAEs) was comparable between the CTB and PBO groups across cohorts. Of the 36 PT, 25 (CTB 67%, PBO 78%) reported 65 TEAEs. Nausea, headache, and fatigue were the most reported TEAEs in CTB-treated participants. TEAEs occurring in > 1 PT receiving CTB are listed in Table 2. No significant changes were noted in clinical labs, ECGs, or vital signs, and there were no serious AEs, drug-related discontinuations, or deaths.

**Conclusion:**

Cis-CTB exposure (AUC) increased in a dose proportional manner. Single doses of CTB up to 1200 mg and multiple doses of CTB up to 400mg doses q8h for 10 days were safe and well tolerated.

**Disclosures:**

**Mary Beth Dorr, PhD**, Merck: Stocks/Bonds (Public Company)|Pfizer: Stocks/Bonds (Public Company)|Venatorx: Stocks/Bonds (Private Company) **Carlos Fernando de Oliveira, MD, PhD, MS**, Venatorx Pharmaceuticals: Stocks/Bonds (Private Company) **Kathryn Lowe, MS**, Venatorx Pharmaceuticals: Stocks/Bonds (Private Company) **Gregory A. Winchell, PhD**, Certara: Advisor/Consultant|Merck: Stocks/Bonds (Public Company)|Venatorx: Advisor/Consultant **Paul McGovern, MD**, Venatorx Pharmaceuticals, Inc.: employee|Venatorx Pharmaceuticals, Inc.: Stocks/Bonds (Private Company)

